# Is metformin use associated with cognitive, physical and cardiovascular outcomes in older adults with type 2 diabetes? A secondary analysis of the SCOPE multicentre prospective cohort study

**DOI:** 10.1136/bmjopen-2025-114393

**Published:** 2026-11-09

**Authors:** Johanna Wallensten, Johan Ärnlöv, Lucia Muglia, Luca Soraci, Fabrizia Lattanzio, Paolo Fabbietti, Rada Artzi-Medvedik, Itshak Melzer, Francesc Formiga, Rafael Moreno-Gónzalez, Tomasz Kostka, Agnieszka Guligowska, Zuzanna Chrzastek, Malgorzata Piglowska, Regina Roller-Wirnsberger, Gerhard Wirnsberger, Lisanne Tap, Francesco Mattace-Raso, Axel C Carlsson

**Affiliations:** 1 Department of Clinical Sciences, Danderyd Hospital, 18288 Stockholm, Sweden; 2 Academic Primary Health Care Centre, Region Stockholm, Stockholm, Sweden; 3 Division of Family Medicine and Primary Health Care, Department of Neurobiology, Care Sciences and Society, Karolinska Institute, 17177 Stockholm, Sweden; 4 Center for Biostatistic and Applied Geriatric Clinical Epidemiology (IRCCS INRCA), National Institute of Health and Science on Ageing, INRCA-IRCCS, Cosenza, Italy; 5 Italian National Research Center on Aging (IRCCS INRCA), INRCA-IRCCS, Ancona, Italy; 6 Recanati School for Community Health Professions at the faculty of Health Sciences, Ben-Gurion University of the Negev, Beer-Sheva, Israel; 7 Geriatric Unit, Internal Medicine Department, Systemic Diseases and Ageing Group, Cardiovascular, Respiratory and Systemic Diseases and Cellular Aging Program, Institut d'Investigació Biomèdica de Bellvitge (IDIBELL), L’Hospitalet de Llobregat, Hospital Universitari de Bellvitge, Bellvitge, Spain; 8 Department of Geriatrics, Healthy Ageing Research Centre, Medical University of Lodz, Pomorska 247/249, 92-209 Lodz, Poland; 9 Department of Internal Medicine, Research Unit on Old Age Medicine and Lifelong Health, Medical University of Graz, Graz, Austria; 10 Department of Internal Medicine, Section of Geriatric Medicine, University Medical Center Rotterdam, Erasmus MC, Rotterdam, Netherlands

**Keywords:** Diabetes Mellitus, Type 2, Cognitive dysfunction, Aging, CLINICAL PHARMACOLOGY, Death, Diabetic nephropathy & vascular disease

## Abstract

**Abstract:**

**Objectives:**

Type 2 diabetes (T2D) is common in older adults and often coexists with reduced kidney function, which increases the risk of cognitive decline and cardiovascular events. The aim was to examine the association between metformin use and cognitive function, physical function, disability, cardiovascular events and all-cause mortality in older adults with T2D.

**Design:**

Secondary prospective analysis of the Screening for Chronic Kidney Disease among Older People across Europe (SCOPE) study (conducted between August 2016 and August 2018), a multicentre 2-year study in which metformin exposure was not randomised. Regression models were used to evaluate associations between metformin use and study outcomes.

**Setting:**

Community-based multicentre study conducted across Europe.

**Participants:**

The analysis included 412 community-dwelling older adults (≥75 years) with T2D enrolled in the SCOPE study (mean age 80.2 years, 46.1% women).

**Outcome measures:**

Cognitive function assessed by the Mini-Mental State Examination (MMSE), physical performance assessed by the Short Physical Performance Battery, handgrip strength, functional status and disability (basic activities of daily living (BADL) and instrumental activities of daily living (IADL)), cardiovascular events and mortality.

**Results:**

Metformin users maintained stable MMSE scores while a slight decrease in MMSE score was observed in non-users. Significant baseline imbalances existed: non-metformin users had lower estimated glomerular filtration rate (44.8 vs 56.3 mL/min) and higher heart failure prevalence (28.9% vs 13.5%). At 2 years, metformin users maintained stable MMSE scores, while non-users showed a slight decrease (0.0 vs −0.7; p<0.001); however, this statistical difference is very small and unlikely to be clinically meaningful in the short term. Additionally, metformin use was associated with a lower risk of cardiovascular events and cardiovascular mortality at 2 years (HR: 0.50; 95% CI 0.30 to 0.84). There were no significant differences in physical function and handgrip strength, BADL or IADL.

**Conclusions:**

Metformin use was associated with preserved cognitive performance over 2 years, while a slight decline was observed among non-users. Additionally, metformin was associated with lower risk of cardiovascular events and mortality. However, given the 2-year duration of the study, further research is needed to confirm these findings, particularly in relation to cognitive decline and dementia, which have a long prodromal phase.

**STRENGTHS AND LIMITATIONS OF THIS STUDY**

- This study included community-dwelling adults aged 75 years and older with type 2 diabetes from a large multicentre European cohort.
- The analyses accounted for kidney function and multiple clinical confounders relevant to metformin prescribing in older adults.
- The prospective design enabled assessment of cognitive, physical, cardiovascular and mortality outcomes over 2 years of follow-up.
- As an observational study, residual confounding and confounding by indication cannot be excluded.
- Information on changes in metformin treatment during follow-up was not available.

## Introduction

 Diabetes is a rapidly growing global health challenge, with more than 800 million people having a diagnosis.^1^ Type 2 diabetes (T2D), which accounts for 95% of all diabetes cases, has a prevalence exceeding 20% among individuals aged 70 and older.^2 3^ This rise, driven by ageing populations and lifestyle changes, underscores urgent public health concerns.^2^ Type 2 diabetes is also a leading risk factor for disability and cardiovascular events.^4^

Metformin, a first-line treatment for T2D, is widely recognised as an effective and safe antihyperglycaemic agent with notable metabolic benefits.^5^ Its primary mechanism involves reducing hepatic glucose production.^6 7^ Additionally, metformin increases glucose uptake in skeletal muscles, reduces the effects of glucagon in the liver, enhances autophagy function in pancreatic β-cells and alters the gut microbiome, potentially leading to glucose excretion via faeces.^8^ Such mechanisms may be relevant not only for glycaemic regulation, but also for broader health outcomes in older people, as both muscle glucose uptake and gut microbiota have been linked to physical frailty and sarcopenia.^9 10^ Furthermore, metformin decreases appetite and body weight by elevating levels of the growth/differentiation factor 15,^11^ a factor that has been associated with frailty in older people.^12 13^ Beyond this, metformin exhibits diverse possible therapeutic effects on longevity, mediated by the induction of apoptosis, inhibition of proliferation of malignant cells and the provision of cardiovascular and neuroprotective benefits.^5^

Metformin’s cardiovascular and neuroprotective properties have garnered significant attention. While a meta-analysis shows that metformin reduces cardiovascular events and mortality in patients with T2D, conflicting evidence suggests that combining it with other glucose-lowering agents may increase mortality risks.^14 15^ Similarly, its potential cognitive benefits, including reduced dementia risk in older patients with T2D at higher doses, are counterbalanced by studies showing adverse effects, such as exacerbated cognitive decline and pathological markers in Alzheimer’s models, particularly in older populations.^16 17^ Taken together, the available evidence is inconsistent, highlighting the need for further studies in older adults with T2D, particularly those with chronic kidney disease (CKD), who are often underrepresented in previous research. However, there were different study designs and experiments behind these findings making additional studies warranted.

Metformin is predominantly metabolised renally, hence sufficient renal function is critical for its safe use. It is usually contraindicated in advanced renal impairment (estimated glomerular filtration rate (eGFR) <30 mL/min × 1.73 m²) and requires dose adjustments or caution according to kidney function. Reduced kidney function is common among older adults and has been associated with multiple adverse outcomes, including cognitive impairment,^18^ frailty, cognitive decline, cardiovascular disease and increased mortality; careful T2D management in this group is vital.^19–23^

Research into metformin’s cognitive and cardiovascular benefits in CKD is challenging. While its effects in this population remain unclear, discontinuing metformin in patients with T2D with CKD may increase cardiovascular and renal risks.^24^ Emerging evidence suggests that use below an eGFR of 30 mL/min × 1.73 m² could provide cardiovascular, renal and mortality benefits, though these must be weighed against the risk of lactic acidosis.^24^ Making adjustments due to kidney function is of importance.

Metformin has previously been found to improve physical activities such as gait speed, hand grip strength and Short Physical Performance Battery (SPPB); however, findings have also shown no effect.^25 26^ The improved hand grip strength in the metformin group may be due to its potential muscle-protective effects but could also be due to its effect on inflammation and oxidative stress.^26^

Given that older adults with T2D are at heightened risk of multimorbidity, functional decline, cognitive impairment and cardiovascular events, it is crucial to examine how metformin use relates to these outcomes. Understanding these associations may clarify the broader role of metformin in promoting healthy ageing beyond its glycaemic effects. The aim of this study was to examine the association between metformin use and cognitive function, physical function, disability, cardiovascular events and all-cause mortality in older individuals with T2D after 2 years of follow-up, in analyses adjusted for kidney function and other common risk factors.

## Method

The present study was a secondary prospective analysis performed within the database of the ‘Screening for Chronic Kidney Disease among Older People across Europe’ (SCOPE) study.^27^ The SCOPE study (European grant agreement number 436849) is a multicentre 2-year prospective cohort study involving older adults 75 years old and over, living independently in the community and attending the outpatient clinics in participating institutions in Austria, Germany, Israel, Italy, the Netherlands, Poland and Spain.^27^ Clinical trial number is not applicable as the study was observational. Participants aged 75 years or more attending outpatient services at participating institutions from August 2016 to August 2018 were asked to participate and reinvestigated after 2 years.

Participants were excluded if they were patients with end-stage renal disease or dialysis, history of solid organ or bone marrow transplantation, active malignancy within 24 months prior to screening or metastatic cancer, life expectancy less than 6 months, severe cognitive impairment (Mini-Mental State Examination (MMSE) <10) and patients unwilling to provide consent and those who cannot be followed-up. Medical history and use of medication classified according to the WHO definition were collected during all face-to-face visits, self-reported and with data from medical records.

### Study population

This substudy of SCOPE was designed to address two primary aims through distinct analyses. The first aim was to explore the association between metformin use and changes in cognitive function, physical performance and handgrip strength over a 24-month period (n=412), and is shown as a flow chart in figure 1. To examine this, we included individuals with a diagnosis of T2D according to clinical records or those using antidiabetic medications at baseline (Anatomical Therapeutic Chemical (ATC) Codes: A10B–A10X) with available data 24 months after recruitment and have complete data available for the SPPB,^28^ handgrip strength and MMSE^29^ and assessments at both baseline and the 24-month follow-up.

**Figure 1:**
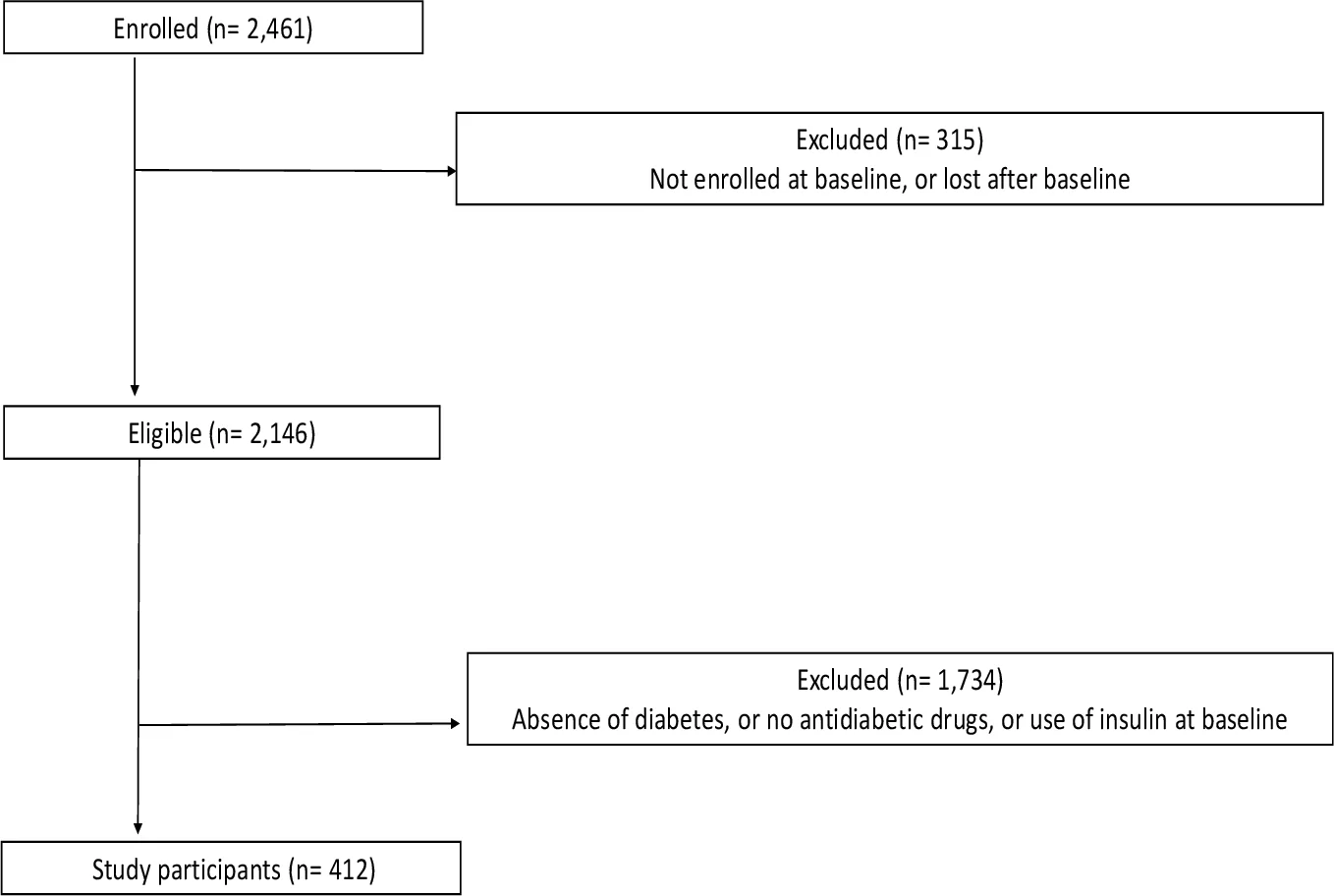
Flowchart of the inclusion criteria for the present study.

The second aim was to investigate the association between metformin use and the incidence of cardiovascular events and cardiovascular mortality. Additionally, all subjects included in this analysis had complete follow-up data available after recruitment.

Individuals using insulin were excluded from the study.

### Exposure

Metformin use at baseline was self-reported or identified in the medical records at the hospital. The participants were stratified based on use of metformin with or without other concomitant antidiabetic drugs at study entry, Yes or No.

### Outcome measures and assessment tools

The *MMSE* was used to examine cognitive status.^29^ MMSE is primarily a screening test for cognitive disorders, not a quantitative test of cognitive function. Yet, MMSE evaluates domains such as orientation, short-term memory, attention and calculation, language comprehension and visuospatial abilities through a series of 11 structured questions. The total score ranges from 0 to 30, with higher scores indicating better cognitive performance.^29 30^ However, MMSE is not a diagnostic tool on its own; scores had been interpreted within the broader clinical context, considering factors such as education level, language proficiency and cultural background, to ensure an accurate assessment and appropriate management.

Physical function and disability were assessed using the SPPB, handgrip strength, basic activities of daily living (BADL) and instrumental activities of daily living (IADL). The *SPPB* is a validated tool for assessing lower extremity physical performance and predicting all-cause mortality.^31 32^ It evaluates physical function through three tests: balance, walking speed and the ability to rise from a chair. Each test is graded on a scale from 0 (lowest performance) to 4 (highest performance), resulting in a total score that can range from 0 to 12. A higher score indicates better physical function.^28^

Grip strength is a marker for overall health and sarcopenia that can be used for identifying older adults at risk of poor health.^33^ It is predictive for all-cause mortality, future functional decline, cognitive issues and hospitalisation-related problems.^33^

*BADL* and *IADL* are used for rating of self-reported disability.^34 35^ BADL assesses self-care activities such as dressing and feeding, IADL assesses more complex activities required for independent living, such as shopping, housekeeping and medication management.

Cardiovascular events were based on clinical records and included atrial fibrillation, congestive heart failure, stroke/transient ischaemic attack (TIA), angina/myocardial infarction (MI) and death from cardiovascular causes after the baseline investigation.

*Mortality, all-cause,* was based on clinical records and defined as death after the baseline investigation.

### Covariates (potential confounders)

*Age* was reported in years at the baseline investigation. *Sex* was obtained from the medical record, *eGFR* was estimated using creatinine-based eGFR calculated using the Berlin Initiative Study 1 equation, baseline *blood glucose* level, *number of prescribed medications* at baseline, self-reported baseline *DPP4-inhibitor* use, as well as history of cardiovascular comorbidities: *atrial fibrillation*, *heart failure*, *hypertension*, *stroke/TIA* and *MI/angina*.

### Patient and public involvement

Patients and members of the public were not involved in the design, conduct, reporting or dissemination plans of this research.

### Statistical analysis

Descriptive statistics were used to characterise the population, and Mann-Whitney U test was used to determine the unadjusted differences in individuals with and without metformin at baseline as well as over 24 months.

In an effort to address potential bias the following models were tested: analysis of covariance (ANCOVA) was used to determine the association between metformin and differences in several variables over 24 months and Cox regression was used to estimate the HR of CV events and cardiovascular mortality. Cox and ANCOVA models were adjusted for the following potential confounders: age, sex, eGFR, baseline blood glucose level, number of prescribed medications, baseline DPP4-inhibitor use, atrial fibrillation, heart failure, hypertension, stroke/TIA and MI/angina and outcome variables at baseline. Proportional hazards were checked using time-dependent metformin interaction among the covariates of the two Cox models.

Statistical significance was set at p<0.05. All statistical analyses were performed with SPSS V.24 (SPSS, Chicago, Illinois, USA).

## Results

Baseline characteristics of individuals with T2D were compared based on the occurrence of metformin use (table 1). Among the 412 individuals included in the analysis, 260 were treated with metformin (63.1%). Key baseline differences were observed between users and non-users. Those treated with metformin had higher eGFR (56.3 vs 44.8 mL/min/1.73 m², p<0.001) but a lower MMSE score (27.7 vs 28.9, p<0.001). Additionally, heart failure was more prevalent in individuals not receiving metformin (28.9% vs 13.5%, p<0.001).

**Table 1:** Baseline characteristics of the included participants

	Non-metformin use group (n=152)	Metformin use group (single+combinations) (n=260)	P value
Age (years)	80.0 (5.7)	79.0 (5.0)	0.483
Gender (woman)	73 (48.0)	117 (45.0)	0.552
Education (years)	11.0 (5.0)	12.0 (7.0)	0.124
BMI (kg/m²)	28.8 (5.7)	28.3 (5.0)	0.536
eGFR BIS (ml/min)	44.8 (12.9)	56.3 (19.9)	<0.001***
ACR (mg/g)	18.4 (83.9)	13.0 (33.9)	0.040*
MMSE score	28.9 (2.0)	27.7 (2.3)	<0.001***
Glucose (mg/dL)	116.5 (42.5)	127.0 (44.0)	0.082
Number of prescribed medications	8.0 (4.0)	8.0 (4.0)	0.251
Atrial fibrillation	34 (22.4)	40 (15.4)	0.075
Congestive heart failure	44 (28.9)	35 (13.5)	<0.001***
Hypertension	135 (88.8)	228 (87.7)	0.734
Stroke/TIA	10 (6.6)	37 (14.2)	0.018*
Angina/myocardial infarction	36 (23.7)	43 (16.5)	0.075
Glibenclamide or glimepiride	34 (22.4)	50 (19.2)	0.446
Pioglitazone	2 (1.3)	2 (0.8)	0.585
DPP4-inhibitors	36 (23.7)	25 (9.6)	<0.001
GLP-1 analogues	1 (0.7)	3 (1.2)	0.620
SGLT2-inhibitors	3 (2.0)	2 (0.8)	0.281

Data were expressed by number (percentage) or median (IQR). *=p<0.05; **=p<0.01; ***=p<0.001.

ATC codes for the medication: glibenclamide and glimepiride (A10BB)**,** pioglitazone (A10BG)**,** DPP4-inhibitors (A10BH)**,** GLP-1 analogues (A10BJ)**,** SGLT2-inhibitors (A10BK).

ACR, albumin-to-creatinine ratio; ATC, Anatomical Therapeutic Chemical; BIS, Berlin Initiative Study; BMI, body mass index; eGFR, estimated glomerular filtration rate; MMSE, Mini-Mental State Examination; TIA, transient ishaemic attack.

Baseline characteristics of individuals with T2D were also compared based on the occurrence of major cardiovascular events or cardiovascular mortality during follow-up (see online supplemental appendix table 1 in online supplemental material). Key differences in those with events included lower rate of metformin treatment, higher baseline albumin-to-creatinine ratio and more commonly previous angina or MI.

### Unadjusted comparisons

Further analysis compared clinically relevant endpoints at baseline and after 24 months among participants with and without metformin use (table 2). Differences in baseline physical performance, cognitive function and dependency in BADL between the two groups were highlighted, providing insights into the potential impact of metformin on long-term outcomes.

**Table 2:** Characteristics for clinically relevant end points in those alive and attending follow-up after 24 months in patients with type 2 diabetes and chronic kidney disease with and without metformin use

	No metformin (n=152)	Metformin (combinations) (n=260)	Median difference (95% CI)	P value*
SPPB (total score at baseline)	8.0 (4.7)	9.0 (4.0)	−1.0 (−1.0 to 0.0)	0.005**
SPPB delta (24 months score—baseline score)	−1.0 (3.0)	0.0 (3.0)	0.0 (−1.0 to 1.0)	0.952
Handgrip (best score at baseline)	25.5 (17.0)	24.0 (14.0)	1.0 (−1.0 to 3.0)	0.410
Handgrip delta (24 months score—baseline score)	−1.0 (5.0)	−1.0 (6.0)	0.0 (−1.0 to 1.0)	0.812
MMSE (at baseline)	28.9 (2.0)	27.7 (2.3)	1.0 (0.7 to 1.0)	<0.001***
MMSE delta (24 months score—baseline score)	−0.7 (2.6)	0.0 (2.7)	−1.0 (−1.7 to −0.7)	<0.001***
Number of BADL dependency (baseline)	0 (0)	0 (0)	0.0 (0.0 to 0.0)	0.206
BADL dependency delta (24 months score—baseline score)	0 (0)	0 (0)	0.0 (0.0 to 0.0)	0.788
Number of IADL dependency (baseline)	1 (3)	0 (2)	0.0 (0.0 to 0.0)	0.040*
IADL dependency delta (24 months score—baseline score)	0 (0)	0 (1)	0.0 (0.0 to 0.0)	0.283

Data were expressed by median (IQR). Median differences were calculated from individual-level data and may therefore differ from the arithmetic difference between the reported group medians.

*=p<0.05; **=p<0.01; ***=p<0.001.

* Mann Whitney U test.

BADL, basic activities of daily living; IADL, instrumental activities of daily living; MMSE, Mini-Mental State Examination; SPPB, Short Physical Performance Battery.

Cognitive performance, measured by the corrected MMSE, was slightly lower at baseline in the metformin group compared with the non-metformin group (27.7 vs 28.9; p<0.001). However, over 24 months, MMSE scores remained stable in the metformin group, while they declined slightly in the non-metformin group (0.0 vs −0.7; p<0.001).

Physical performance, measured by SPPB total score, reflected better physical performance in individuals on metformin compared with those without (nine in the metformin group vs eight in the non-metformin group, p=0.005). However, no significant differences were observed in changes over 24 months (mean change 0.0 vs −1.0 p=0.952). Baseline handgrip strength did not significantly differ between individuals with metformin use and individuals without (24.0 vs 25.5; p=0.410), nor did changes over time (−1.0 vs −1.0; p=0.812).

For dependency in BADL no significant differences were found at baseline or in changes over time between the groups.

Number of dependencies in IADL was slightly lower at baseline in the metformin group compared with the non-metformin group (0 vs 1; p=0.040).

All-cause mortality was higher in patients without metformin use and associated medication (10.5% vs 4.6%, p=0.021). Cardiovascular mortality followed a similar pattern, with rates of 5.9% vs 1.2% (p=0.005) (Data not shown in tables). Additionally, the combined incidence of CV events and cardiovascular mortality was 25.7% in patients without metformin use or combinations versus 15.0% in those with metformin (p=0.008).

### Adjusted regression analyses

**Table 3:** The association between metformin use and changes in MMSE over 24 months, and HRs for cardiovascular events and cardiovascular mortality over 2 years, with adjustments for relevant variables.

Dependent variable (at 24 months)	Group comparison	Adjusted coefficient (B)	95% CI	P value
Cognitive function				
MMSE score	Metformin vs non-use	0.82	0.23 to 1.42	0.007**
Physical performance				
SPPB score	Metformin vs non-use	0.41	−0.17 to 0.99	0.164
Grip strength	Metformin vs non-use	−0.11	−1.94 to 1.72	0.907
Functional autonomy				
BADL Dependences	Metformin vs non-use	−0.03	−0.18 to 0.11	0.648
IADL Dependences	Metformin vs non-use	0.14	−0.21 to 0.49	0.428

**Event outcome**	Group comparison	HR	95% CI	P value
2 years overall mortality	Metformin vs non-use	0.58	0.22 to 1.57	0.287
2 years cardiovascular events and cardiovascular mortality composite	Metformin vs non-use	0.50	0.30 to 0.84	0.008*

Linear regression and Cox regression model, respectively. Multivariable ANCOVA models for 24-month functional outcomes and Cox proportional hazards regression for cardiovascular events according to metformin use.

The models were adjusted for age, sex, estimated glomerular filtration rate, baseline blood glucose level, number of prescribed medications at baseline, self-reported baseline DPP4-inhibitor use, as well as history of cardiovascular comorbidities: atrial fibrillation, heart failure, hypertension, stroke/TIA and myocardial infarction/angina and relative outcome score at the baseline.

*=p<0.05; **=p<0.01; ***=p<0.001.

ANCOVA, analysis of covariance; BADL, basic activities of daily living; IADL, instrumental activities of daily living; MMSE, Mini-Mental State Examination; SPPB, Short Physical Performance Battery; TIA, transient ischaemic attack;

The linear regression model showed that metformin use was associated with maintained MMSE scores over 24 months, whereas MMSE scores declined among individuals not using metformin, after adjustments for potential confounders listed in table 1.

According to the Cox regression model (table 3) metformin use was associated with a lower hazard of cardiovascular events and cardiovascular mortality (HR 0.53; 95% CI 0.32 to 0.87; p=0.012) after adjustments for the same covariates. The association with all-cause mortality was non-significant.

No significant associations were observed for SPPB, handgrip strength, BADL or IADL in the adjusted analyses. Proportional hazards were checked using time-dependent metformin interaction among the covariates of the two Cox models and since the interaction term was not statistically significant (p>0.05), the assumption was met and the standard Cox proportional hazards model with constant hazards over time was maintained.

## Discussion

The main findings of the study show a possible association of metformin on cognitive function and cardiovascular outcomes, whereas no impact on physical performance and functional dependency was detected.

Metformin use was associated with maintained cognitive performance, assessed through MMSE. At baseline, the metformin use group had slightly lower MMSE scores, possibly reflecting differences in health profiles. To better understand this relation, we performed a longitudinal analysis. Notably, cognitive performance scores remained stable in the metformin use group but declined slightly among non-users over 24 months. In adjusted linear regression models the differences favouring metformin remained significant. These findings suggest a potential association between metformin use and cognitive function. However, the possibility of regression toward the mean cannot be excluded, as the observed stability in the metformin use group could partially reflect statistical tendencies rather than a true protective effect. The results remained significant after adjustments and are consistent with prior studies suggesting that metformin may reduce the risk for dementia and improve cognitive outcomes in T2D populations.^16^ Some conflicting evidence however exists, including animal studies showing negative effects of prolonged metformin use in older mice, such as impaired memory, learning difficulties, exacerbated cognitive decline and increased pathological markers in Alzheimer’s models.^17^ Nevertheless, these findings imply a possible important effect of metformin on cognitive decline and a need to better understand this effect in experimental longitudinal studies. Such studies should then also use more detailed assessments of general and executive cognitive functions.

Previous studies have reported a dose-dependent protective effect of metformin on cognitive status.^16^ Although this study lacked detailed dosage data, it is noteworthy that metformin monotherapy was associated with significant benefits. Possible explanations include differences in underlying mechanisms—such as 5’ adenosine monophosphate-activated protein kinase activation,^36^ mitochondrial function or inflammation^37^ as well as treatment adherence or patient characteristics. These factors warrant further investigation in future studies.

Baseline physical performance, as measured by the SPPB score, was slightly higher in the metformin use group. However, no significant differences were observed in the trajectory of physical decline over 24 months. It is important to note that the observed baseline differences could reflect a selection bias, as individuals with better initial physical performance may have been more likely to receive metformin treatment. Moreover, handgrip strength, an indicator of muscle strength and sarcopenia, showed no significant differences at baseline or over time, indicating that metformin’s benefits may not extend to physical performance or muscle strength in this group of older individuals with T2D.

The observed potential protective cardiovascular effect associated with metformin use aligns with prior research, including a meta-analysis demonstrating that metformin reduces cardiovascular events and cardiovascular mortality in patients with T2D.^14^ Notably, this study did not replicate earlier findings suggesting increased cardiovascular and all-cause mortality when metformin is combined with other antidiabetic drugs.^15^ However, those with combination therapy were few and no significant differences were found between the groups regarding cardiovascular risks in such combinations.

This study has several limitations that should be acknowledged. First, baseline differences between the metformin and the non-metformin groups, including differences in MMSE scores, kidney function and cardiovascular comorbidity burden, and glycaemic status, indicate the potential for confounding by indication and residual confounding. Although the analyses were adjusted for clinically relevant covariates, unmeasured factors may still have influenced the observed associations. Still, the association remained significant after adjustment for relevant laboratory values, kidney function and comorbidities.

Specifically, the marked baseline imbalance in heart failure prevalence (28.9% in non-users vs 13.5% in metformin users) strongly reflects confounding by indication. While heart failure was included as an individual covariate in all multivariable models to minimise bias, residual confounding cannot be entirely eliminated. This limitation may lead to an overestimation of the protective cardiovascular effects attributed to metformin, and the HRs must be interpreted with caution.

Second, the observational design restricts the ability to draw definitive conclusions about causality, and information on initiation, discontinuation or changes in metformin treatment during follow-up was not available. Despite these limitations, our findings are consistent with previous studies in cohorts with younger patients with T2D, where most individuals did not have CKD.^16^

As CV events and mortality in this study were based on clinical documentation, not rigorous adjudication, errors may have contributed to observed group differences. Lastly, the study population primarily comprised relatively well-functioning individuals, who had normal cognition at baseline per MMSE score, which may limit the generalisability of findings to those with more advanced frailty or comorbidities. However, the present study expands previous findings of cognitive,^16^ as well as cardiovascular protective benefits of metformin^14^ in older populations with T2D. Perhaps the fact that metformin should be used with caution in individuals with reduced kidney function has discouraged clinicians from prescribing it to patients with T2D and concomitant CKD. The present study does, however, suggest that there may be clinical benefits. Unfortunately, the length of this follow-up was only 2 years and further studies with longer follow-up are warranted. Additional larger studies may also stratify findings based on cardiovascular disease at baseline; as we adjusted for cardiovascular comorbidities to maximise the sample size. Changes in diabetes management may limit the generalisability of these findings to current clinical practice.

## Conclusion

Metformin use was associated with maintained cognitive function and lower cardiovascular event rates over 24 months in older adults with T2D. However, given baseline differences and the observational nature of the study, these associations should be interpreted with caution. No significant effects were observed for physical performance or handgrip strength. Further research is needed to clarify the potential benefits of metformin in older, more diverse populations with T2D.

## Supplementary material

10.1136/bmjopen-2025-114393 online supplemental file 1

## Data Availability

Data are available upon reasonable request.
