# Supplementary material for: Is metformin use associated with cognitive, physical and cardiovascular outcomes in older adults with type 2 diabetes? A secondary analysis of the SCOPE multicentre prospective cohort study
Source: BMJ Open. 2026 Nov 9;16(11):e114393. doi: 10.1136/bmjopen-2025-114393 (PMC13756269; doi:10.1136/bmjopen-2025-114393)
Supplement: Supplementary file 1 [file bmjopen-2025-114393.reviewer_comments.pdf]

PEER REVIEW HISTORY

BMJ Open publishes all reviews undertaken for accepted manuscripts. Reviewers are asked to complete a checklist review form and are provided with free text boxes to elaborate on their assessment. These free text comments are reproduced below.

ARTICLE DETAILS

Title (Provisional)

Is metformin use associated with cognitive, physical and cardiovascular outcomes in older adults with type 2 diabetes? A secondary analysis of the SCOPE multicentre prospective cohort study

Authors

Wallensten, Johanna; Ärnlöv, Johan; Muglia, Lucia; Soraci, Luca; Lattanzio, Fabrizia; Fabbietti, Paolo; Artzi-Medvedik, Rada; Melzer, Itshak; Formiga, Francesc; Moreno-Gónzalez, Rafael; Kostka, Tomasz; Guligowska, Agnieszka; Chrzastek, Zuzanna; Piglowska, Malgorzata; Roller-Wirnsberger, Regina; Wirnsberger, Gerhard; Tap, Lisanne; Mattace-Raso, Francesco; Carlsson, Axel C

VERSION 1 - REVIEW

**Reviewer**	**1**
**Name**	**Herbert, Laura L.**
**Affiliation**	**University of South Carolina, College of Nursing**
**Date**	**30-Dec-2025**
**COI**	**None**

This paper is well written. Suggestions are included for improved clarity and flow for the reader.

The line numbers start over on each page, so page numbers are included. The page numbers used are the ones provided by the PDF file, not the page numbers on the authored manuscript. Also, line numbers do not align, so the line number provided is an estimate.

Page 3, line 3 – Correct the uppercase letters in the title so all major words are capitalized. The title includes cognition, physical performance, CV events, and mortality in the title, but you also evaluated changes in handgrip strength.

There are discrepancies in the stated aims:

Page 6, line 10 – In the abstract, you only have one aim that includes cognitive decline, physical function, CV outcomes, and mortality, but on page 11, line 21, two aims are listed. Again, there is no mention of handgrip strength as an aim until page 11.

Page 6, Line 23 – duplicate term in the same sentence – SCOPE

Page 8, line 57 – the paper discusses metformin and its benefits and possible adverse effects in the next few paragraphs, but the flow can be improved for the reader. Perhaps consider adding a transition paragraph in between lines 23-52 and 57 that discusses the need for this cohort study to clarify the juxtaposition of contrasting studies. This lets the reader know that the next few paragraphs are going to present conflicting results (e.g., neuroprotective vs. exacerbates cognitive decline)

Page 8, line 18 – In the abstract (page 6, line 8), it is mentioned that T2D coexists with reduced kidney function and this increases the risk of cognitive decline, but in the introduction paragraph there is no mention of the kidney disease/cognitive decline connection. Consider including.

Page 12, line 13 are listed as Outcomes, but I recommend listing this section as Tools. When I read "outcomes" I expected to read the outcomes of the results.

Change for consistent use of the acronym throughout the paper – BADL or bADL? IADL or iADL?

I also recommend keeping the same order as you previously presented the information. On page 6, line 10 in the abstract the order is cognitive decline (MMSE), physical function (SPPB), CV events, and mortality. There are additional tools discussed here (grip strength, bADL, and iADL) that are not mentioned in the abstract or in the aims. This is confusing for the reader.

Additionally, each tool needs to be described in detail to include its reliability and validity, especially the bADL and iADL. Were both tools used? What information did each tool provide?

Page 14, line 3 – was written consent received for the original study or for this cohort study?

The results section was well written. I recommend the paper present information in a consistent order starting with cognitive performance, physical performance, CV events, etc.

Reviewer 2

Name Boucaud-Maitre, Denis

Affiliation University of the French West Indies University and Hospital Library of Guadeloupe

Date 09-Apr-2026

COI None

---

1. This study is affected by several major sources of bias that are only superficially acknowledged and not adequately addressed by the authors. The comparison groups are not comparable at baseline, particularly with respect to cardiovascular history, eGFR, and

glycemic status, leading to substantial confounding by indication. At a minimum, a propensity score–based approach should have been implemented. Furthermore, the use of linear regression models to assess longitudinal changes in outcomes such as MMSE and SPPB is not appropriate. Mixed-effects models should be used for repeated measures. If only two time points are available (baseline and 2-year follow-up), an ANCOVA approach would be more appropriate and statistically more powerful than analyzing change scores.

2. In the linear regression model used for MMSE, it is unclear why the authors adjusted for baseline MMSE, given that the analysis was conducted on change scores (ΔMMSE). This approach is methodologically questionable.

3. The between-group difference in MMSE is clinically negligible (a 0.7-point difference at 2 years). Therefore, I do not agree with the authors' conclusion that "metformin may be associated with a slight cognitive benefit in older adults with type 2 diabetes."

4. The abstract lacks essential information, including participants' age, sex distribution, and key baseline clinical differences between groups, which constitute a major limitation of the study.

5. The study period is not explicitly reported. It must be inferred from the references that the data were likely collected more than a decade ago, involving antidiabetic therapies that are no longer representative of current clinical practice.

6. Throughout the manuscript, the authors imply that the study specifically pertains to older adults with chronic kidney disease (as stated in the key messages), which is misleading. No specific analyses are presented for participants with mild-to-moderate renal impairment.

**Reviewer**	**3**
**Name**	**Dack, Kyle**
**Affiliation**	**University of Bristol, MRC Integrative Epidemiology Unit**
**Date**	**09-May-2026**
**COI**	**None**

Statistical review summary:

The core design and statistical analysis use reasonable methods and I have no major suggestions or queries. Most of my suggestions are minor and relate to clarifying elements of the methods and results. The most substantial is to confirm that model assumptions were tested and summarise the findings in the results.

Abstract:

1. There is a HR reported for CV events or mortality of 0.53. This differs from Table 3, where the HR is 0.50. The 95% CI is also slightly different.

Methods:

2. Study dates and follow-up: The present manuscript is missing the study dates, which could be reported early in the methods.

3. Exposure: What time period does the exposure relate to - e.g. was it present day metformin use? It would be helpful to report the specific question used to determine its use because this can impact the interpretation.

4. MMSE: Are there any prior studies which support the score range interpretation, or were these determined by this study?

5. The outcomes bADL and iADL could be described in slightly more detail, e.g. what is being measured, the range or units of the outcome.

6. Mortality: There is a risk that readers could confuse this with cardiovascular mortality with is mentioned in other sections, and this could be avoided by clarifying it is all-cause mortality.

7. Statistical analysis: The modelling strategy and analysis overall looks reasonable. Given that the data comes from multiple centers, there could be systematic differences across countries or clinics which would impact the assumption of independent observations in standard linear regression. This could be checked by comparing exposure and outcome values per center, or estimating the ICC.

Both linear and Cox's regression involve data assumptions and I suggest adding a brief line for how these were checked (e.g. plotting residuals, testing proportional hazards), and reporting the findings in the results.

Results:

8. "reflected better physical performance in individuals on metformin compared to those without (9 vs. 8, p = 0.005)." this and later sentences could clarify that the reported values are the means. For the first comparison, it could also help readers to make the comparison groups explicit, e.g. (9 metformin vs 8 without metformin, p = 0.005). I don't think this is necessary for all the results (except where the order changes), but if presented for the first comparison it would ensure readers understand the format.

9. The results would be clearer if subheadings were used to signpost which part of the analysis is being reported. This would ensure that sections such as paragraph 4 are not misinterpreted as reflecting adjusted results. Currently there is more space dedicated to the unadjusted results than the adjusted model results (which are less vulnerable to confounding bias), and adding more statistical information about the model results could be valuable. e.g. the coefficient and 95% CI for MMSE, all-cause mortality. The model results for the outcomes SPPB, grip strength, and ADL are not commented on, and could be briefly reported to give a full overview of the model findings.

10. Table 2. Are columns 2 and 3 presenting medians or means? In some cases the median difference does not seem aligned with the presented differences in these columns, perhaps a footnote could clarify how they are calculated?

11. Table 3: The statistic for mortality is described as an RR in the table title, but HR in the table itself. I think HR is more accurate. I notice this was also described as an RR in the statistical analysis section of the methods.

12. Table 3: there are a few differences between the adjustment set reported in the footnote, and that in the methods (e.g. ACR, angina, glucose).

Discussion:

13. No suggestions - the interpretation looks aligned with the design and results, and limitations are described well.

VERSION 1 - AUTHOR RESPONSE

Reviewer: 1

Dr. Laura L. Herbert, University of South Carolina

Comments to the Author:
This paper is well written. Suggestions are included for improved clarity and flow for the reader.

Author response: We thank the reviewer for the positive comments and constructive suggestions. We have revised the manuscript accordingly and provide detailed responses to each comment below.

The line numbers start over on each page, so page numbers are included. The page numbers used are the ones provided by the PDF file, not the page numbers on the authored manuscript. Also, line numbers do not align, so the line number provided is an estimate.

Page 3, line 3 – Correct the uppercase letters in the title so all major words are capitalized. The title includes cognition, physical performance, CV events, and mortality in the title, but you also evaluated changes in handgrip strength.

Author response: We thank the reviewer for the careful reading of our manuscript. We have revised the title and corrected the capitalization.
The title has been revised to: "Is metformin use associated with cognitive, physical and cardiovascular outcomes in older adults with type 2 diabetes? A secondary analysis of the SCOPE multicentre prospective cohort study"

There are discrepancies in the stated aims:

Page 6, line 10 – In the abstract, you only have one aim that includes cognitive decline, physical function, CV outcomes, and mortality, but on page 11, line 21, two aims are listed. Again, there is no mention of handgrip strength as an aim until page 11.

Author response: We thank the reviewer for this important observation. We agree that the study aims should be described consistently throughout the manuscript. To address this, we have revised the wording of the study aim in both the Abstract and the Introduction to ensure that all investigated outcomes are clearly and consistently specified.
The study aim in both the Abstract and the Introduction has been revised to consistently include cognitive function, physical function, disability, cardiovascular outcomes, and mortality. Handgrip strength is now considered part of the physical function domain throughout the manuscript. The aim reads as follows now: "The aim was to examine the the association between metformin use and cognitive function, physical function, disability, cardiovascular events, and all-cause mortality in older adults with T2D."

Page 6, Line 23 – duplicate term in the same sentence – SCOPE

Author response: We thank the reviewer for noting this error. The duplicate term has been removed.

Page 8, line 57 – the paper discusses metformin and its benefits and possible adverse effects in the next few paragraphs, but the flow can be improved for the reader. Perhaps consider adding a transition paragraph in between lines 23-52 and 57 that discusses the need for this cohort study to clarify the juxtaposition of contrasting studies. This lets the reader know that the next few paragraphs are going to present conflicting results (e.g., neuroprotective vs. exacerbates cognitive decline)

Author response: We thank the reviewer for this helpful suggestion. To improve the flow of the Introduction and provide a clearer rationale for the study, we have added text highlighting the inconsistent findings reported in previous studies regarding the cardiovascular and cognitive effects of metformin. This transition now better contextualizes the conflicting evidence and underscores the need for further investigation in older adults with type 2 diabetes and impaired kidney function.
Changes made in manuscript: A transition sentence emphasizing the inconsistent evidence and the resulting need for further research has been added to the Introduction. "Taken together, the available evidence is inconsistent, highlighting the need for further studies in older adults with T2D, particularly those with chronic kidney disease, who are often underrepresented in previous research. "

Page 8, line 18 – In the abstract (page 6, line 8), it is mentioned that T2D coexists with reduced kidney function and this increases the risk of cognitive decline, but in the introduction paragraph there is no mention of the kidney disease/cognitive decline connection. Consider including.

Author response: We have added a statement and supporting reference describing the association between chronic kidney disease and cognitive impairment in older adults;

"Reduced kidney function is common among older adults and has been associated with with multiple adverse outcomes, including cognitive impairment [18],.."

Page 12, line 13 are listed as Outcomes, but I recommend listing this section as Tools. When I read "outcomes" I expected to read the outcomes of the results.
Change for consistent use of the acronym throughout the paper – BADL or bADL? IADL or iADL?
I also recommend keeping the same order as you previously presented the information. On page 6, line 10 in the abstract the order is cognitive decline (MMSE), physical function (SPPB), CV events, and mortality. There are additional tools discussed here (grip strength, bADL, and iADL) that are not mentioned in the abstract or in the aims. This is confusing for the reader.
Additionally, each tool needs to be described in detail to include its reliability and validity, especially the bADL and iADL. Were both tools used? What information did each tool provide?

Author response: We thank the reviewer for these helpful comments. We have revised this section to improve clarity and consistency. The section heading has been modified to better reflect that it describes assessment instruments and outcome measures. Acronyms have been standardized throughout the manuscript. We have also expanded the descriptions of BADL and IADL, including the information provided by these assessments and their role as measures of disability and functional independence.
In addition, the order of presentation has been revised to better align with the study aims and outcomes described elsewhere in the manuscript. The methods section on these instruments now reads:

"Outcome measures and assessment tools
The MMSE was used to examine cognitive status [29]. MMSE is primarily a screening test for cognitive disorders, not a quantitative test of cognitive function. Yet, MMSE evaluates domains such as orientation, short-term memory, attention and calculation, language comprehension, and visuospatial abilities through a series of 11 structured questions. The total score ranges from 0 to 30, with higher scores indicating better cognitive performance [29, 30]. However, MMSE is not a diagnostic tool on its own; scores had been interpreted within the broader clinical context, considering factors such as education level, language proficiency, and cultural background, to ensure an accurate assessment and appropriate management.

Physical function and disability were assessed using the SPPB, handgrip strength, BADL, and IADL.."

Page 14, line 3 – was written consent received for the original study or for this cohort study?

Author response: We thank the reviewer for this comment. We have clarified that written informed consent was obtained from all participants at enrolment in the original SCOPE study, from which the present secondary analysis was conducted. It now reads:

"Written informed consent was obtained from all participants at inclusion in the original SCOPE study, from which the present secondary analysis was derived."

The results section was well written.

Author response: We thank the reviewer for the kind remarks.

I recommend the paper present information in a consistent order starting with cognitive performance, physical performance, CV events, etc.

Author response: Excellent suggestion. To improve consistency throughout the manuscript, we have revised the order of presentation in the Results section. The results are now presented in the same sequence as the study aims and outcome measures, beginning with cognitive outcomes, followed by physical function and disability outcomes, and finally cardiovascular events and mortality.

Reviewer: 2

Dr. Denis Boucaud-Maitre, University of the French West Indies University and Hospital Library of Guadeloupe, University of the French West Indies and Guiana

Comments to the Author:

1. This study is affected by several major sources of bias that are only superficially acknowledged and not adequately addressed by the authors. The comparison groups are not comparable at baseline, particularly with respect to cardiovascular history, eGFR, and glycemic status, leading to substantial confounding by indication. At a minimum, a propensity score–based approach should have been implemented. Furthermore, the use of linear regression models to assess longitudinal changes in outcomes such as MMSE and SPPB is not appropriate. Mixed-effects models should be used for repeated measures. If only two time points are available (baseline and 2-year follow-up), an ANCOVA approach would be more appropriate and statistically more powerful than analyzing change scores.

Author response: We thank the reviewer for these important comments. We acknowledge the potential for confounding by indication and residual confounding, which are inherent limitations of observational studies. To address this, the analyses were adjusted for a range of clinically relevant covariates, including: "***Age***, ***Sex***, Glomerular filtration rate ***eGFR*** estimated using creatinine-based eGFR calculated using the Berlin Initiative Study 1 (BIS1) equation, baseline ***MMSE*** , baseline ***blood glucose*** level, ***number of prescribed medications*** at baseline, self-reported basline ***DPP4-inhibitor*** use, as well as history of cardiovascular comorbidities: ***atrial fibrillation***, ***heart failure***, ***hypertension***, *stroke/TIA* and ***myocardial infarction/angina***."

Regarding the statistical approach, we agree that ANCOVA could be considered better than regression models for our work. So, also given the observational nature of the study and the availability of only two assessment time points, we changed Table 3 with ANCOVA method.

The limitation section in the discussion now reads:
"This study has several limitations that should be acknowledged. First, baseline differences between the metformin and the non-metformin groups, including differences in MMSE scores, kidney function, and cardiovascular comorbidity burden, and glycaemic status, indicate the potential for confounding by indication and residual confounding. Although the analyses were adjusted for clinically relevant covariates, unmeasured factors may still have influenced the observed associations. Still, the association remained significant after adjustment for relevant laboratory values, kidney function, and comorbidities.
Second, the observational design restricts the ability to draw definitive conclusions about causality, and information on initiation, discontinuation, or changes in metformin treatment during follow-up was not available. Despite these limitations, our findings are consistent with previous studies in cohorts with younger patients with T2D, where most individuals did not have CKD [16]."

2. In the linear regression model used for MMSE, it is unclear why the authors adjusted for baseline MMSE, given that the analysis was conducted on change scores (ΔMMSE). This approach is methodologically questionable.

Author response: We thank the reviewer for this important comment. Now we changed linear regression models with ANCOVA, so for example, in the model with outcome "MMSE at 24-months, you will see MMSE at baseline as covariate.

3. The between-group difference in MMSE is clinically negligible (a 0.7-point difference at 2 years). Therefore, I do not agree with the authors' conclusion that "metformin may be associated with a slight cognitive benefit in older adults with type 2 diabetes."

Author response: We thank the reviewer for this important observation. We agree that the observed difference in MMSE scores was modest and should be interpreted cautiously. To avoid overstating the clinical significance of this finding, we have revised the wording in the Abstract and Discussion. The manuscript now refers to preserved cognitive performance rather than a cognitive benefit.

4. The abstract lacks essential information, including participants' age, sex distribution, and key baseline clinical differences between groups, which constitute a major limitation of the study.

Author response: We thank the reviewer for the valuable additions. The Abstract has been revised to provide additional information regarding the study population, including age and sex distribution. The Participants section now reports the mean age and proportion of women

in the study cohort. Baseline differences between groups remain described in detail in the main manuscript and are accounted for in the adjusted analyses.

5. The study period is not explicitly reported. It must be inferred from the references that the data were likely collected more than a decade ago, involving antidiabetic therapies that are no longer representative of current clinical practice.

Author response: We thank the reviewer for this comment. The study recruitment and follow-up period have now been reported more explicitly in the Methods section. We have also added a limitation acknowledging that the study was conducted before the widespread use of newer glucose-lowering therapies, which may affect the generalisability of the findings to current clinical practice. The limitation reads:

"Changes in diabetes management may limit the generalisability of these findings to current clinical practice."

6. Throughout the manuscript, the authors imply that the study specifically pertains to older adults with chronic kidney disease (as stated in the key messages), which is misleading. No specific analyses are presented for participants with mild-to-moderate renal impairment.

Author response: We thank the reviewer for this observation. We agree that the present study was not a dedicated CKD subgroup analysis. To avoid overstating the role of CKD in the study population, we have revised the wording in the manuscript and removed statements that could imply that the study specifically examined older adults with chronic kidney disease throughout the manuscript.

Reviewer: 3

Dr. Kyle Dack, University of Bristol

Comments to the Author:
Statistical review summary:
The core design and statistical analysis use reasonable methods and I have no major suggestions or queries. Most of my suggestions are minor and relate to clarifying elements of the methods and results. The most substantial is to confirm that model assumptions were tested and summarise the findings in the results.

Abstract:
1. There is a HR reported for CV events or mortality of 0.53. This differs from Table 3, where the HR is 0.50. The 95% CI is also slightly different.

Author response: We thank the reviewer for identifying this inconsistency. We have checked the reported values and corrected the Abstract to ensure consistency with the results presented in Table 3.

Methods:
2. Study dates and follow-up: The present manuscript is missing the study dates, which could be reported early in the methods.

Author response: We thank the reviewer for this suggestion. The study recruitment and follow-up period have now been reported explicitly in the Methods section to improve clarity. We have added the following:

"Participants aged 75 years or more attending outpatient services at participating institutions from August 2016 to August 2018 were asked to participate and reinvestigated after 2 years."

3. Exposure: What time period does the exposure relate to - e.g. was it present day metformin use? It would be helpful to report the specific question used to determine its use because this can impact the interpretation.

Author response: We thank the reviewer for this helpful comment. We have clarified that metformin exposure was defined according to current metformin use at baseline (study entry), based on self-report and/or review of medical records. The Methods section has been revised accordingly.

4. MMSE: Are there any prior studies which support the score range interpretation, or were these determined by this study?

Author response: We agree that the MMSE was used in the present study as a continuous measure of cognitive performance rather than as a diagnostic tool based on predefined score categories. To avoid confusion, we have simplified the description of the MMSE in the Methods section, removed the score classifications, and added a reference supporting its use as a screening instrument for cognitive impairment.

5. The outcomes bADL and iADL could be described in slightly more detail, e.g. what is being measured, the range or units of the outcome.

Author response: We thank the reviewer for this helpful suggestion. We have expanded the descriptions of BADL and IADL in the Methods section to provide additional detail regarding the domains assessed. The revised text now clarifies that BADL assesses basic self-care activities, whereas IADL assesses more complex activities required for independent living.

6. Mortality: There is a risk that readers could confuse this with cardiovascular mortality with is mentioned in other sections, and this could be avoided by clarifying it is all-cause mortality.

Author response: Good suggestion. To improve clarity and avoid confusion with cardiovascular mortality, we have revised the manuscript to consistently refer to this outcome

as all-cause mortality where appropriate.

7. Statistical analysis: The modelling strategy and analysis overall looks reasonable. Given that the data comes from multiple centers, there could be systematic differences across countries or clinics which would impact the assumption of independent observations in standard linear regression. This could be checked by comparing exposure and outcome values per center, or estimating the ICC.
Both linear and Cox's regression involve data assumptions and I suggest adding a brief line for how these were checked (e.g. plotting residuals, testing proportional hazards), and reporting the findings in the results.

Author response: We thank the reviewer for this comment. We checked proportional hazards using time-dependent metformin interaction among the covariates of the 2 Cox models. Since the interaction term was not statistically significant ($p > 0.05$), the assumption was met, and the standard Cox proportional hazards model with constant hazards over time was maintained. We have added this sentence to the section Statistical analysis;
Proportional hazards were checked using time-dependent metformin interaction among the covariates of the 2 Cox models.

Results:
8. "reflected better physical performance in individuals on metformin compared to those without (9 vs. 8, p = 0.005)." this and later sentences could clarify that the reported values are the means. For the first comparison, it could also help readers to make the comparison groups explicit, e.g. (9 metformin vs 8 without metformin, p = 0.005). I don't think this is necessary for all the results (except where the order changes), but if presented for the first comparison it would ensure readers understand the format.

Author response: We thank the reviewer for this helpful suggestion. We have revised the Results section to better clarify the comparison groups in the reported values.

9. The results would be clearer if subheadings were used to signpost which part of the analysis is being reported. This would ensure that sections such as paragraph 4 are not misinterpreted as reflecting adjusted results. Currently there is more space dedicated to the unadjusted results than the adjusted model results (which are less vulnerable to confounding bias), and adding more statistical information about the model results could be valuable. e.g. the coefficient and 95% CI for MMSE, all-cause mortality. The model results for the outcomes SPPB, grip strength, and ADL are not commented on, and could be briefly reported to give a full overview of the model findings.

Author response: We thank the reviewer for this helpful suggestion. To improve clarity, we have revised the Results section to more clearly distinguish between the unadjusted comparisons (Table 2) and the adjusted regression analyses (Table 3). We have also expanded the description of the adjusted model results in the text, including outcomes for which no significant associations were observed, to provide a more complete overview of the findings.

10. Table 2. Are columns 2 and 3 presenting medians or means? In some cases the median difference does not seem aligned with the presented differences in these columns, perhaps a footnote could clarify how they are calculated?

Author response: We thank the reviewer for this helpful observation. The values presented in the first two columns are medians with interquartile ranges, as indicated in the table footnote. To improve clarity, we have expanded the footnote to explain how the reported median differences were calculated and why these values may not correspond exactly to the arithmetic difference between the reported group medians.

11. Table 3: The statistic for mortality is described as an RR in the table title, but HR in the table itself. I think HR is more accurate. I notice this was also described as an RR in the statistical analysis section of the methods.

Author response: We thank the reviewer for identifying this inconsistency. We agree that hazard ratio (HR) is the correct term for estimates derived from Cox regression models. The terminology has now been revised throughout the manuscript, including the Methods section and Table 3, to ensure consistency.

12. Table 3: there are a few differences between the adjustment set reported in the footnote, and that in the methods (e.g. ACR, angina, glucose).

Author response:
We thank the reviewer for identifying these inconsistencies. We have made sure that the models are presented correctly both in the methods and in the Tables.

Discussion:
13. No suggestions - the interpretation looks aligned with the design and results, and limitations are described well.

Author response: We thank the reviewer for the positive comments and for noting that the interpretation and discussion are aligned with the study design and results.

VERSION 2 - REVIEW

**Reviewer**	**1**
**Name**	**Herbert, Laura L.**
**Affiliation**	**University of South Carolina, College of Nursing**
**Date**	**20-Jul-2026**

COI

Thank you for addressing all of my concerns. Well written manuscript.

**Reviewer**	**2**
**Name**	**Boucaud-Maitre, Denis**
**Affiliation**	**University of the French West Indies University and Hospital Library of Guadeloupe**
**Date**	**20-Jul-2026**
**COI**	

I remain of the opinion that this study is not sufficiently robust and that the risk of bias is too substantial to support publication in BMJ Open.

1. Although heart failure appears to have been included as an adjustment variable (presumably entered as an individual covariate), the marked baseline imbalance in heart failure prevalence (29% vs. 13%) raises serious concerns about residual confounding. Please clarify whether heart failure was indeed modeled as an individual covariate. In addition, the authors should justify why a propensity score-based approach (e.g., matching or inverse probability weighting) or another method to improve baseline comparability was not used, and discuss the potential impact of this imbalance on the interpretation of the cardiovascular outcomes.

2. The abstract should clearly report the important baseline differences between the two groups, particularly regarding eGFR and heart failure prevalence. It should also state that the observed difference in MMSE change was very small and is unlikely to be clinically meaningful. Presenting a statistically significant result without acknowledging its limited clinical relevance may be misleading.

3. The abstract should also clearly specify the study period. This information is essential because diabetes management strategies, including the use and positioning of glucose-lowering therapies, have substantially evolved over time. Therefore, the findings may not fully reflect current clinical practice in diabetology, and this limitation should be appropriately acknowledged.

VERSION 2 - AUTHOR RESPONSE

Reviewer 2 (Comment 1): Although heart failure appears to have been included as an adjustment variable (presumably entered as an individual covariate), the marked baseline imbalance in heart failure prevalence (29% vs. 13%) raises serious concerns about residual confounding. Please clarify whether heart failure was indeed modeled as an individual

covariate. In addition, the authors should justify why a propensity score-based approach (e.g., matching or inverse probability weighting) or another method to improve baseline comparability was not used, and discuss the potential impact of this imbalance on the interpretation of the cardiovascular outcomes.

Authors response: We thank the Reviewer for this insightful methodological comment. We confirm that history of congestive heart failure was modeled strictly as an individual, dichotomous covariate (Yes/No) within all multivariable ANCOVA and Cox proportional hazards regression models. It was not bundled into a composite score, ensuring its specific variance was accounted for mathematically. We also gave serious consideration to propensity score matching (PSM) and inverse probability weighting (IPW) during the design phase. However, given our highly specific target population (≥75 years with T2D, n=412), implementing PSM would have led to a substantial loss of unmatched participants, particularly from the non-metformin group (n=152) which carried a heavy comorbidity burden. Such a reduction would severely compromise the statistical power needed to evaluate 2-year cardiovascular events and mortality. Standard multivariable regression adjustment was therefore selected to maximize the utilization of the available cohort data.

We fully agree that despite tight multivariable adjustment, the baseline imbalance (higher heart failure and lower eGFR in non-users) flags a clear confounding by indication (i.e., sicker patients being less likely to receive metformin). We have expanded our Discussion (Limitations section) to explicitly address how this residual confounding might overstate the apparent cardiovascular benefits of metformin: "Specifically, the marked baseline imbalance in heart failure prevalence (28.9% in non-users vs. 13.5% in metformin users) strongly reflects confounding by indication. While heart failure was included as an individual covariate in all multivariable models to minimize bias, residual confounding cannot be entirely eliminated. This limitation may lead to an overestimation of the protective cardiovascular effects attributed to metformin, and the hazard ratios must be interpreted with caution."

Reviewer 2 (Comment 2): The abstract should clearly report the important baseline differences between the two groups, particularly regarding eGFR and heart failure prevalence. It should also state that the observed difference in MMSE change was very small and is unlikely to be clinically meaningful. Presenting a statistically significant result without acknowledging its limited clinical relevance may be misleading.

Authors response: We completely agree with the Reviewer. Statistical significance does not automatically equate to clinical relevance, especially over a short 2-year window. We have modified the Abstract to report the baseline differences (eGFR, heart failure) and tempered the interpretation of the MMSE changes. "Results (in Abstract section): Significant baseline imbalances existed: non-metformin users had lower eGFR (44.8 vs 56.3 mL/min) and higher heart failure prevalence (28.9% vs 13.5%). At 2 years, metformin users maintained stable MMSE scores, while non-users showed a slight decrease (0.0 vs -0.7; $p<0.001$); however, this statistical difference is very small and unlikely to be clinically meaningful in the short term. Additionally, metformin use was associated with..."

Reviewer 2 (Comment 3): The abstract should also clearly specify the study period. This information is essential because diabetes management strategies, including the use and positioning of glucose-lowering therapies, have substantially evolved over time. Therefore, the findings may not fully reflect current clinical practice in diabetology, and this limitation should be appropriately acknowledged.

Authors response: This is an excellent point. The positioning of SGLT2-inhibitors and GLP-1 receptor agonists has indeed shifted radically since the study data collection. We have added the exact study period to the Abstract: "Design: Secondary prospective analysis of the Screening for CKD among Older People across Europe [SCOPE] study (conducted between August 2016 and August 2018)...". In the discussion section, we already wrote: "Changes in diabetes management may limit the generalisability of these findings to current clinical practice.".
