# Supplementary material for: Is metformin use associated with cognitive, physical and cardiovascular outcomes in older adults with type 2 diabetes? A secondary analysis of the SCOPE multicentre prospective cohort study
Source: BMJ Open. 2026 Nov 9;16(11):e114393. doi: 10.1136/bmjopen-2025-114393 (PMC13756269; doi:10.1136/bmjopen-2025-114393)
Supplement: Supplementary file 2 [file bmjopen-16-11-s001.docx]

**Appendices Table 1. Baseline characteristics**

**Baseline characteristics in individuals with type 2 diabetes and chronic kidney disease, stratified by major cardiovascular event or cardiovascular mortality during follow up.**

|  | | | **2-years cardiovascular events + cardiovascular mortality** | | **p** |
| --- | --- | --- | --- | --- | --- |
|  |  |  |  | |  |
|  |  |  | No (n=334) | Yes (n=78) |  |
|  |  | Age, median (IQR) | 80.0 (5.0) | 79.5 (6.3) | 0.139 |
|  |  | Gender (female), n (%) | 154 (46.1) | 36 (46.2) | 0.994 |
|  |  | Metformin current (single + association), n (%) | 221 (66.2) | 39 (50.0) | 0.008** |
|  |  | GLP1+DPP4 current, n (%) | 51 (15.3) | 14 (17.9) | 0.559 |
|  |  | Glucose, median (IQR) | 123.0 (44.0) | 124.5 (39.5) | 0.739 |
|  |  | eGFR BIS, unit median (IQR) | 53.7 (20.7) | 51.3 (20.3) | 0.125 |
|  |  | Albumin-to-creatinine ratio (ACR) , median (IQR) | 13.0 (37.2) | 20.7 (77.4) | 0.029* |
|  |  | MMSEscore , median (IQR) | 28 (2.7) | 28.1 (2.0) | 0.071 |
|  |  | Number of current drugs, median (IQR) | 8.0 (4.0) | 8.0 (3.0) | 0.176 |
|  |  | Atrial fibrillation | 58 (17.4) | 16 (20.5) | 0.514 |
|  |  | Congestive heart failure | 68 (20.4) | 11 (14.1) | 0.206 |
|  |  | Hypertension | 294 (88.0) | 69 (88.5) | 0.914 |
|  |  | Stroke/Tia | 34 (10.2) | 13 (16.7) | 0.105 |
|  |  | Angina/Myocardial infarction | 56 (16.8) | 23 (29.5) | 0.010* |

*= p<0.05; **= p<0.01; ***= p<0.001.

List of abbreviations: IQR inter quartile range, Albumin-to-Creatinine Rratio (ACR), Berlin Initiative Study (BIS), Dipeptidyl peptidase 4 inhibitors (DPP4), Glucagon-like peptide 1 (GLP-1), Estimated Glomerular Filtration Rate (eGFR), Berlin Initiative Study (BIS), Mini-Mental State Examination (MMSE),Transient ishemic attack (TIA).
